# An Essay on the Philosophy of Medical Science

**Published:** 1845-04

**Authors:** 


### An Essay on the Philosophy of Medical Science. By *Elisha Bartlett*, M.D. Philadelphia, 1844, 8vo. pp. 310.

The disposition to eschew hypothesis as a guide for medical practice, and to fall back upon diligent observation, is rapidly augmenting in this country; and we should feel at a loss at the present time to point to any dominant theory assuming the empire over the mind of observers similar to that once possessed by the systems of Darwin, Cullen, or Brown. This is a matter for congratulation; for although almost any system may furnish some useful practical indications, yet the evil it produces by its exclusiveness, and by diverting attention from the true path of inquiry, infinitely preponderates over any resulting benefit. The object of the present Essay is to demonstrate that all Science, properly so called, must have its foundation laid, and in fact consists in, the careful observation of facts, and not in *à priori* or hypothetical reasoning. It is still more important that fanciful hypothesis should not be our guide in medical science than it is in physical science, inasmuch as the practical results of erroneous views are of so much more vital consequence. In the first part of his work, Dr. Bartlett sets forth the characters of true physical science; and in the second, exhibits the precisely similar nature of medical science, as well as some of the obstacles which oppose themselves to its completeness, and to its pursuit. In each of these there is matter that will prove interesting to our readers.

### Part I. The Philosophy of Physical Science.

The purport of the entire work may be said to be briefly stated in the first proposition advanced by the author, that "*all physical science consists in ascertained facts, or phenomena, or events; with their relations to other facts, or phenomena, or events; the whole classified, and arranged.*" The following observations upon the partial apprehension of this statement, even by those who profess to reject all *á priori* reasoning, are deserving of attention.

"I believe, nevertheless, it is true, that there has always been, and that there still is, in the minds of most men, and in those of philosophical thinkers, a somewhat imperfect, or confused, apprehension of its doctrines. I do not think that its truth is seen and felt, as it should be, in the simplicity, the purity, and the absoluteness, which belong to it. The confusion, to which I allude, is this. There seems to be a common feeling, that the facts, phenomena, and events, with their relationships, classified and arranged, constitute, not the entire science, to which they belong, but only the *foundation* of the science. There is a feeling that these facts and relations are to be used as elements, out of which the science is to be built up, or constructed, by what is called *inductive reasoning*. The feeling implies, and the avowed doctrine growing out of it often asserts, that the science *is in this subsequent process of reasoning*, and not in the facts, themselves, and their relationships. We are constantly told, that the facts are to be used as *materials*, to be sure; that it is not safe to take for our materials any thing but facts; that they constitute the basis of every science; but, after all this, the essential condition and constitution of the science is often placed, more in the process of reasoning, as it is called, than in the facts and their relationships. Now what I wish to insist upon is this : that the science *is in the facts and their relationships, classified and arranged, and in nothing else.* The science, thus constituted, is, so far, complete no proof of inductive reasoning, or of any other reasoning, no act of the mind can add any thing to what has already been done. * * * * * * * Words are things ; and I cannot doubt, that much obscurity and confusion would be removed from our conceptions of the nature of the philosophy of science, if this long-abused term, *inductive reasoning,* could be suffered to disappear from the language of science and philosophy; and if, for the indefinite and shadowy ideas, which it so often expresses, or attempts to express, could be substituted those, which are so clearly and obviously contained in the phraseology,—*the classification and arrangement of phenomena and their relationships.*"

As illustrative of this view of the nature of the philosophy of physical science, Dr. Bartlett cites the phenomena of *gravitation,* which, observed and classified, form the science completely, and exclusively of any subsequent process of reasoning.

That a knowledge of these various facts or phenomena, with their relationships, can only be acquired by *observation,* or experience, is admitted in a general way frequently, without a full appreciation of the consequences of such admission. The necessity of each class of phenomena or relationships being separately submitted to this process is not sufficiently allowed, and the possibility of deducing the existence of certain of them from the knowledge of the existence of certain others, is too readily admitted. After giving various illustrations of the independence of these classes of phenomena of each other, as regards the proof of their existence, the author observes.

" There is another seeming qualification of the doctrine, that I am endeavouring to illustrate, about which it may be requisite to say a few words. It has often been alleged, for instance, that Sir I. Newton inferred, by a process of *à priori* reasoning, the combustibility of the diamond, before such combustibility had been demonstrated by observation. But what did Newton really do in this case ? Manifestly this and nothing more. A relationship had already been noticed between two certain properties or phenomena,—at least in many bodies ; to wit, their refractive power and their combustibility. Newton's reasoning, as it is called, consisted, simply, in the suggestion, or conjecture, that this relationship might be absolute and universal : and, if so, that the diamond would prove to be combustible. The only reasoning in the case consisted in the application to new circumstances of an assumed relationship. It has been said of Fresnel, that he 'proved by a most profound mathematical inquiry, *à priori,*' the existence of certain subtle properties of polarized light. But here, again, what did Fresnel really do ? He showed, by the agency of his mathematical calculations, that certain relationships of light, assumed or ascertained by observation, in certain conditions, must, if these relationships were true and genuine, exist, also, in all other identical conditions. He showed that, if certain modifications of light, wrought in its properties, by the action of Iceland spar, during its passage through this substance, were dependent upon certain peculiarities in its crystalline structure, then the same modification must be produced in other substances, identical in these peculiarities of structure with the Iceland spar. He applied, merely, and generalized, by means of his calculations, a phenomenon, or relationship of light, already ascertained by direct observation." 25.

A *law* or principle of physical science is defined as consisting " *in a*

*rigorous and absolute generalization of the facts, phenomena, events, and relationships, by the sum of which, science is constituted; and in nothing else. It is identical with the universitality of a phenomenon, or the invariableness of a relationship.*" It is this universitality and unchangeableness of the tendency of all substances, left at liberty, to approach each other that constitutes the law of gravitation. So, too, with the refrangibility of the rays of light, and various other well-ascertained phenomena.

*The Nature and Value of Hypothesis.*—The author defines hypothesis as "*an attempted explanation, or interpretation of the ascertained phenomena and relationships, constituting science: and it is nothing else. It consists in an assumption, or a supposition, of certain other unascertained and unknown phenomena. It does not constitute an essential element of science. All science is absolutely independent of hypothesis.*" The desire of explaining the nature of the various phenomena which are only partially known and appreciable by us, has possessed the inquiring spirit of man in all times: but its exercise must not be confounded with and considered as part and parcel of science. The various speculations upon the ultimate constitution of matter, even such as have all the plausibilities and probabilities in their favour, as that of the Atomic Theory, are at best but *conjectural*, having numerous incongruities to contend with, and liable to be dissipated as the field of knowledge enlarges: and yet the facts to be explained, and the co-ordination of phenomena remain immutable. Thus, the facts and science of optics remain unchanged, although the Newtonian hypothesis respecting their nature is abandoned by nearly all enquirers.

A supposed use of hypothesis is thus commented upon.

"There is one very common feeling in regard to these interpretations and explanations, which is, that they render the phenomena, to which they are applied, more intelligible—more easily comprehended and understood than they otherwise would be. It seems to me, that there is some fallacy in this feeling, or, at least, that its alleged value is exaggerated. We shall find, I think, on a close examination of the matter, that the difficulty to which I refer, is only changed in the place which it occupies, by these explanations; that it is neither removed, nor very materially diminished, by them."

This opinion is illustrated by inquiring as to what extent is the comprehension of the phenomena of gravitation really rendered more easy by Newton's assumption of the intervention of an invisible æther. The explanation is nowise facilitated by the supposed addition of this material but invisible substance, one difficulty only being substituted for another. The same observation applies to the various theories of light, electricity, chemical attraction, &c.

Dr. Bartlett, however, by no means denies that hypotheses, properly employed, may have their great uses.

"Without qualifying, in any degree, the doctrine I have been endeavouring to elucidate, that all science is independent of hypothesis, I am quite willing to admit, that hypothesis has often been of service to science, in suggesting, guiding, and directing its researches. I am willing to go farther than this, as has already been intimated, and to admit, at least the possibility, in some instances, that the researches thus suggested and directed, may lead ultimately, to the positive demonstration of the assumed phenomena, constituting the theory. I am willing to admit with Professor Whewell (the speculative tendencies of whose mind are very evident in all his writings) the great difficulty, perhaps the impossibility, in many cases, of forming any definite conception of phenomena, or reasoning upon them, without resorting to some hypothetical machinery, for the purpose of expressing their nature and relations. But, after all, I cannot avoid repeating the conviction, that an undue importance, and a false position, is still very generally assigned to these interpretations. The old and illegitimate usurpation of power by the IDEAL PHILOSOPHY, in the empire of science, is even yet only partially destroyed; and the reign of EXPERIENCE, with that divine right, and absolute dominion, which constitute her inalienable prerogatives, has been only partially established. It is important to observe, farther, that the aids and uses, which may really be derived from hypotheses, will in no way be diminished, but increased rather, by assigning to them the subordinate character and station, which they ought always to occupy. If this is done, while their ability to advance the progress of science will not be in any degree lessened, their mischievous tendencies in obscuring its perceptions, and in leading it astray, will be neutralized." 48.

As a makeweight against the authority of Sir John Herschell and Mr. Whewell, the two great advocates for an extended use of hypotheses, the author cites the opinions of Newton and Davy deprecating their undue influence.

Dr. Bartlett furnishes the following definition of a *theory*.

"Theory is one of two things, according to the manner in which the word has been used. It is either a generalization of phenomena, and relationships, and in this case, identical with a law, or principle, of science; or, it is an attempted explanation of phenomena, and relationships, through the intervention of other assumed and unascertained phenomena, and relationships, and, in this case, identical with hypothesis."

*Classification* of phenomena and their relationships is essential to the constitution of science. Certain groups, possessing identity or similarity, require to be arranged together, in marked contradistinction to others, evincing dissimilarities, and in proportion as this is accomplished is the classification a natural one. All is confusion until this is effected; for the mere heterogeneous accumulation of facts may be an unavoidable preliminary, but it is their due arrangement and classification alone that can be said to constitute science.

### PART II. THE PHILOSOPHY OF MEDICAL SCIENCE.

If the temptation to pass from the true path of inquiry and deduction proves often too great for the cultivators of physical science, where so much is known, and so many relationships incontrovertibly established; we may believe that, in the investigation of the science of life, in which the facts acquired are so much fewer in number, and their relations to each other far less obvious, the resort to *à priori* reasoning and hypothesis will be of still more frequent occurrence.

"The feeling has been much more common in medical, than in physical science, that although facts and their relations might, indeed, and must constitute the *foundation* of the science, the science still consisted in something more than these facts and relations;—that, *upon* these latter the science itself was to be somehow built up by that magical and creative process of the mind,—that evil genius of medical science, called, indeed, *induction,* but differing, when stripped of its disguises, in no single function or attribute, from that speculation, the place of which it professed, with promises as loud and pompous as they have proved to be barren and empty, to occupy. The feeling has been, and still is, as much, almost, since the time of Bacon as before,—that the science is in the *inductive* or *reasoning process, superadded to the facts and their relations,* more than in these latter themselves. Here, at the commencement of this part of my essay, I wish to enter my protest against this doctrine, in all its forms and modifications. I wish to show, that the science of medicine consists in the *phenomena of life with their relationships classified and arranged,*—WHOLLY, ENTIRELY, ABSOLUTELY. I wish to show that these elements constitute, not the foundation upon which, nor the materials, merely with which, the science is to be subsequently constructed, by some recondite and logical process of the reason, —but that they *are* the science, and the whole science, already constructed, and so far completed; and that nothing can be superadded to them, by any act of the mind, which can in any way increase their value, or change their character." 69.

So important do we regard the inculcation of the necessity of the study of facts as the only legitimate foundation of all science, that we have perhaps allowed the author to repeat himself too often in the extracts we have made from his book. We may, however, now state, that we believe he has in some measure raised a phantom for the mere purpose of destroying it, and that a portion of his objections at least are resolvable into a misapprehension of terms. Far be it from us to state that the so-called medical science even of our own day is not disfigured by hasty conclusions and crude hypotheses. That it is so, and must continue to be, until a more laborious system of observation is recognized as essential (which it is however more and more every year) does not admit of doubt: but we are quite at a loss to know how progress is to be effected if the new facts acquired are not to be submitted to the process of *induction.* In what other manner are our principles for the comprehension and treatment of disease to be acquired? In fact, the author, in one proposition of his essay, contradicts the conclusions of another: for, while he repudiates all idea of science being dependent upon induction, he at the same time asserts that the acquisition of facts and phenomena is useless, unless they be submitted to due classification and arrangement. Truly it is so; but this very classification of a large body of facts is but another term for a generalization of them, which generalization is in point of fact the inductive process itself; and no scientific law or principle can possibly be laid down until it has been gone through. The author has had in view the numberless instances of induction from an insufficient number of facts, and the dangerous consequences of reasoning from false analogies: but such perversions as these no-wise justify his indiscriminate and inconsistent condemnation of the process of induction, when legitimately carried out. We recommend to the notice of our readers the most able statement upon this subject we are acquainted with, namely, a Lecture delivered some years since by Dr. Ferguson, upon "*The Method of Induction and its Results on Medical Science.*"*

---

* Appended to his Treatise on Puerperal Fever, 1839.

The author dwells at some length upon the following proposition. "*Each separate class of facts, phenomena and events, with their relationships, constituting as far as they go, medical science, can be ascertained in only one way; and that is by observation, or experience. They cannot be deduced, or inferred, from any other class of facts, phenomena, events, or relationships, by any process of induction or reasoning, independent of observation.*"

The prevalent belief is that so great a connection exists between the different branches of medical science, that the knowledge of the one infers a knowledge of another; whereas the knowledge of each series of phenomena or relationships can only be derived from direct observation of such series. For our knowledge of *Physiology* is not, as it is so often said to be, dependent upon our knowledge of *Anatomy*, beyond at least some appreciation of the general law of final causes, or the adaptation of means to the end. Thus, although from this we learn the skull is designed as a protective case for the brain, no anatomical knowledge, however minute and elaborate, could inform us of the functions of this latter organ, many of which were attributed, by the old *à priori* physiologists, to various other organs of the economy. So, too, *Pathology* is not founded upon *Physiology*, and a knowledge of the one is not deducible from a knowledge of the other. This statement, so contrary to received opinions, is yet strictly true; and, as exemplificatory of it, examine into the phenomena of the most general of all affections—*inflammation*—which are better understood than are those attendant upon many other forms of disease.

"Now, I ask, if any attainable knowledge of the healthy action of the parts, in which this process is seated, could of itself, have led us to a knowledge of that diseased action of the same parts, constituting inflammation? Is there anything, susceptible of being ascertained, in the natural functions of these parts, in the properties, the susceptibilities, the actions of the minute arteries, the minute veins, of the capillaries, of the nervous filaments involved in this morbid process, which could have pre-supposed their liability to this process? Could any knowledge of the former have led, by any course or method of reasoning, independent of observation, to a knowledge, or a prediction, of the latter? Could a knowledge of the one have been *deduced* from a knowledge of the other? Most clearly and indisputably not. * * * * * * Do the natural, the unfelt, the unnoticed actions of these minute vessels and nervous filaments pre-suppose, in any way, their liability to those numerous and complex processes—the contractions and distensions of the vessels—the increased, the diminished, the irregular velocity of the blood—the pain, the heat, the secretions—which enter as elements into this morbid condition? Certainly not. What physiological properties of these minute vessels could have informed us of their power, under any circumstances, to separate the fibrine from the other proximate constituents of the blood, or to secrete pus? Certainly none. So far, then, as the phenomena themselves of inflammation are concerned, I do not see how it is possible, that they should be inferred or deduced from the physiological phenomena of the parts with which they are concerned, or in which they are seated: and I think that an examination of all the other circumstances connected with this morbid condition will serve to elucidate and to strengthen this result. Let us take one of these circumstances,—that of the different degree of liability, in the different organs and tissues of the body, to be affected by this morbid process. This difference is very great. Certain parts and organs are very liable to inflammation; other parts and organs are very little so. Now, is there anything in the physiology of these several parts and organs, from which, by any *à priori* reasoning, these different degrees of liability could have been ascertained? Why are the lungs so frequently, and why is the spleen so rarely, the seat of this pathological process? Why is acute inflammation of the pleura, and the pia mater so common, and acute inflammation of the peritoneum, so uncommon an affection? It will not do to say that these different degrees of liability to this disease can be accounted for by any obvious or appreciable differences in the structure and functions of the organs or tissues in which it is seated. These differences between the peritoneum and the pleura, for instance, are not sufficiently striking to account for the result. Neither will it do to say, as has often been said, that the degree of this liability is in proportion to the importance and functional activity of the different organs and tissues. Let us test the value of this pretended explanation, by a reference to the mucous membrane of the *stomach.* It would be difficult, I think, to find any part of the body, in which, from mere *à priori* reasoning, we should be justified in looking for acute inflammation more frequently than in this. In what part is there greater activity of function? In what part are more important processes carried on? In what part is there a quicker or more delicate susceptibility to impressions? What part is more intimately connected with the other important acts and organs of the body? Is it not the great centre of the organic sympathies? What part is more exposed to the action of irritating substances? And yet, notwithstanding all these apparent, and *à priori* causes of acute inflammation, very few tissues, or organs, of the system are so rarely affected by this morbid process as the mucous membrane of the stomach. Certainly, nothing can show more clearly the utter futility of the attempt to explain the fact of which I am speaking, by referring it to differences in the importance and activity of the functions of the different organs, than this striking exemption of the gastric mucous tissue from attacks of acute inflammation." 96.

The same conclusions are deducible from the examination of the phenomena of any other disease; and the only sense in which anatomical and physiological knowledge may be said to be necessary for the recognition of pathological change, is as serving as a *standard of comparison.*

In like manner, *Etiology* is not deducible from our knowledge of pathology, but from observation alone. Independently of observation, how could pathology, for example, inform us of the influence of age, sex, season, &c., in inducing various forms of disease.

The only solid foundation of *Therapeutics* is also a similar course of independent observation. Writers upon this subject have long been divided into two sects, the *Rationalists* and the *Empirics.* The former profess to derive certain indications for the employment of curative means from the pathological condition of the organ affected, and to offer a satisfactory account of the *modus operandi* of these. The empirics, on the contrary, fall back upon experience and observation, and deny that pathology could ever have alone indicated the means requisite for the control of the various morbid phenomena. To revert to the illustration before advanced; how could the treatment of *inflammation* be deduced from a mere *à priori* consideration of its pathology? for, although in some cases the increased heat might seem at once to suggest the application of cold, and the turgidity of vessels the removal of blood, experience alone could have decided whether these conjectures would prove valid, and has decided, that in numerous cases, their indiscriminate carrying out, under circumstances wherein the apparent pathological condition was the same, would prove mischievous or destructive.

"How could any such reasoning have ever led to the conclusion, that the abstraction of blood from the general circulation would have diminished the intensity, or shortened the duration, or in any way changed the action of this local morbid process? How could any such reasoning have led to a knowledge of the circumstances, in which this abstraction of blood would be followed by beneficial results? How could any such reasoning have led to the knowledge of the relationship, which exists between inflammation and the operation of calomel, antimony, and opium? Could the effects of these substances have been deduced, or inferred, from any knowledge, however accurate, of the phenomena of inflammation?" 112.

Then, again, how could the curative power of arsenic or cinchona in periodic disease have ever been ascertained by other mode than empirical observation; and so on with a whole train of remedies which could never have been suggested by the consideration of pathological conditions alone. Neither can the action of remedies in a diseased state of the economy be inferred from the effects they produce upon it in a state of health; nor can effects which have been produced by these in one class of animals be inferred as certain to occur in animals of another class. Slight analogies may in both cases be suggestive of experiment; but it is by the result of the experiment, and not the probability of the analogy, we must abide.

*Diagnosis.*—Diagnosis is perfect, just in proportion to the perfection of our pathological knowledge; and is the most important element in every therapeutical operation.

"Therapeutics consist in the relationships which exist between pathological conditions and actions, on the one hand, and the articles and agents of the materia medica, on the other. These relationships, like all others, are fixed and invariable. The properties of the articles and agents of the materia medica are easily ascertained. It is not from the difficulty of ascertaining these properties, that the uncertainties of therapeutics arise. These uncertainties grow out of, and rest in, the imperfection of our diagnosis; the incompleteness of our knowledge of pathology. Just in proportion to the perfection and absoluteness of our diagnosis; just in proportion to the completeness of our pathological knowledge, will be the certainty of our therapeutics. All practical medicine depends upon a knowledge of three things, to wit, pathology; the articles or agents of the materia medica; and the relations between these two elements; and nearly all the difficulties, the obscurities, the uncertainties, the imperfections of our pathological knowledge, or, in other words, of our diagnosis." 122.

Dr. Bartlett contrasts the different measure of success which attends our therapeutical procedures in *pleurisy*, in which diagnosis is comparatively so easy and so perfect, with that attendant upon the treatment of the complicated, and often obscure, pathological conditions, constituting typhoid fever.

Diagnosis may be either *nosological* or *therapeutical*. The former of these is that to which the term is usually restricted; and by its aid the different morbid processes are recognized and separated, constituting the various diseases. *Therapeutical* diagnosis is defined by Dr. Bartlett as the distinction between individual diseases, dependent upon their relations to the various agencies of the materia medica.

"The first condition of therapeutical diagnosis is a knowledge of the individual disease: but many other, and frequently much more important, conditions of this diagnosis, are to be found in other circumstances. Amongst these may be mentioned, for the purpose of illustrating my meaning, the following:—the extent and severity of the individual disease—its period—in many cases, its occurrence in a sporadic or an epidemic form—the age of the patient—and the general condition of the patient previous to the attack of the individual disease. These circumstances do not enter into our nosological diagnosis; but they frequently constitute altogether the most important elements in the therapeutical relationships of disease. The nosological diagnosis of acute pneumonia, confined to the lower portion of a single lung, does not differ from that of the same disease, involving the whole of one lung, and the half of the other. But this difference in the extent of the disease will affect very essentially its therapeutical relationships, and the diagnosis depending upon these." 145.

*Laws or Principles of Medical Science.*—While the laws of physical science are *positive* and absolute, those of the science of life are *approximative* only. The relationships of the phenomena in the former may be analysed or separated and ascertained, while in the latter they are too numerous and complicated to admit of any such process. "The sum of the phenomena and relationships in any and in every given instance, is not *constant* and *positive*, but *contingent* and *variable*." Such variableness has however certain fixed limits, within which the law becomes absolute. Unless this were the case, medical science could not be said to exist.

"There is, for instance, a certain number of phenomena and relationships, the sum of which constitutes a disease, to which we give the name of *pleurisy*. This sum or aggregate is not absolute, and uniform, but contingent and variable. No one of these aggregates, constituting the disease, is ever exactly equivalent to another; no two cases of pleurisy are ever precisely identical. Still, the differences between them are not unlimited and indefinite: they are always confined within certain degrees. The resemblances between these individual aggregates are sufficiently fixed and positive, to render them determinate and comparable facts; capable of being used as data, and dealt with in our researches and generalizations, subject to the qualifications already made, as we deal with the data of physical science. The same thing is true of all the other groups of morbid phenomena and relationships, constituting the various individual diseases of the nosology. The sum of these phenomena is more variable and fluctuating in some groups, than in others; our knowledge of these phenomena is more accurate and extensive in some groups, than in others; but the degree of fluctuation is always confined within certain limits, which are susceptible of determinate measurement." 152.

The establishment of a therapeutical or diagnostic principle or law requires, according to the complexity or simpleness of the disease, the observation of a greater or less number of examples. Dr. Bartlett refers, with much satisfaction, to M. *Gavarret's* work on *Medical Statistics*, for a full view of the conditions which it is requisite should be observed, in order that the therapeutical indications deduced from observation, and recorded by numeration, may be trustworthy. Among these are, (1), the necessity of the facts being sufficiently definite to be *comparable*, e. g. in regard to locality, class of persons, hygienic circumstances, &c.: (2), the clearness and positiveness of the diagnosis, and recognition of the important varieties of the affection. In testing modes of treatment, there must not be a *selection* of cases, but the plan must be tried in all that present themselves, distinguishing however the varieties of the disease with the appropriate modifications of treatment; (3), the method of treatment must be defined distinctly and clearly; (4), a principle of treatment can only be deduced from the careful analysis of a *great number* of individual instances, and by an application to the average result of the calculation of probabilities. For want of rigorous attention to all these circumstances, which render cases of disease justly *comparable*, so many instances of opposing therapeutical conclusions, and imaginary cures, are constantly brought before the profession and public.

Advocating as he does the great importance of the numerical method of observation, the author is quite aware of the impossibility of its too rigid application in practice. He says—

"How definitively soever the laws of these phenomena and of these relationships may be settled; the individual instances, or elements, of which they are composed, must still continue fluctuating and indeterminate—always, however, within certain limits—the positiveness of the law cannot apply to the individual instances. The exactness of our appreciation of these instances, and our ability to estimate their precise value and conditions, may be aided by an acquaintance with the law: but this appreciation and estimate must still depend mostly upon the extent and accuracy of our knowledge of the several elements, which unite to make up the individual instances themselves. Thus, although the ascertained law of the ratio of mortality in a given disease, under given circumstances, may assist us in predicting the termination in an individual case; still this prediction must depend in a great degree, upon our knowledge of the fluctuating and variable elements of the case itself. No acquaintance, however perfect, with the laws of pathology and therapeutics, can ever remove, or in any degree diminish, the necessity of a thorough and discriminating study and knowledge of the single instances which unite to make up the materials of the law. Our diagnosis, prognosis, and management of individual cases of disease must depend, not so much upon the laws with which the diseases are concerned, as upon an accurate knowledge of the individual cases themselves; so that no perfection, or absoluteness, of the law, can ever lessen the necessity and importance of sagacity, discrimination, and skill, on the part of the physician, in the practical application of his art." 177.

*Medical Doctrines or Hypotheses.*—The tenour of the observations we have hitherto extracted will prepare the reader to expect the antipathy with which the author regards the various Doctrines which have from time to time swayed the medical world. Any praise which may attach to physical hypotheses as guiding and suggesting research, cannot be bestowed upon these, which have only acted as unmitigated evils, retarding progress, mistyfying that which was obscure, and preventing the employment of many useful practical procedures. The hypotheses in physical science are few and fair to behold, but in the science of life "they are without number; their name is legion; and in most instances they are as remarkable for their complexity, clumsiness, and improbability, as the theories of physical science are for the opposite qualities." Sometimes also they do not seem to possess even the merit of having been framed for the better interpretation of disease, but are the mere results of a disordered imagination. The evil influences these doctrines generate are numerous. They incapacitate a man as a faithful *observer* of disease, and disqualify him as a useful practitioner, swayed as he is by preconceived notions, derived from an imaginary pathology. Even where the hypothesis has been founded upon an observation of the effect of remedies, it has often produced an injurious re-action on the subsequent treatment, as may be observed even in the case of Sydenham himself. It is fortunate that the great body of practitioners has seldom allowed itself to be influenced by hypotheses in its treatment of disease, such influence having been usually confined to their speculative originators and a few zealous followers. Not only do hypotheses exert an ill effect upon the practice of medicine, but they impede its scientific advance, precisely as did the false philosophy impede the march of physical science. Nevertheless the use of theory or hypotheses need not be utterly exploded, provided it be received as mere conjecture and not as an element of science. " One of the finest modern instances that I have met with, of the legitimate and admissible use of hypothesis, and of the exact appreciation and estimate of its true function and position, is to be found in Dr. Holland's remarks ' On the hypothesis of insect life, as a cause of disease.' "

The various " medical doctrines" as Methodism, Cullenism, Brownism, Broussaism, are briefly adverted to and condemned; but the *coup de grace* is inflicted upon hypothesis in an examination of *Homœopathy*, the claims, of which to the rank of a system, Dr. Bartlett maintains, are equal to any of these, as far as legitimate foundation is concerned, although they have been more mixed up with charlatanism. The author's compatriots are treated with no gentler hand than the European speculators, for we have a chapter devoted to the exposure of the errors of Rushism, Cooke-ism, Gallup-ism, and Thomson-ism.

We must extract his remarks upon *Broussais*. After observing, that pre-occupation by hypothesis disqualifies the mind for accurate observation, he goes on to say:—

" I have just mentioned Broussais; and I may add, that nowhere can a more striking exemplification of the influence of which I am speaking, be found, than in the history of his mind. His two great works are the *History of Chronic Inflammations*, and the *Examination of Medical Doctrines*. The former is almost entirely a work of pure observation. The clear-headed, sagacious, observer shines out in every one of its pages: they are all luminous with practical wisdom. I am sure that no man at all capable of appreciating it, can read this book, especially the first parts of it, treating of diseases of the lung and pleura, without feeling, that it proceeded from a mind of extraordinary capacity and strength; and without entire reliance on the accuracy and good faith of the author, as an observer. What precision and positiveness in his diagnosis! What enlarged, but cautious comprehensiveness in his general conclusions! What honesty and frankness in his admission of the frequent impotency of medical art! What admirable tact and discrimination in his selection and use of remedial measures! How clear and sound the philosophy which illumines and binds all this together! How true his appreciation of the emptiness and worthlessness of theoretical speculations; equal almost to that of Newton and Davy! * * * * * * * * * But in his *Examination of Medical Doctrines*, all this is far otherwise. Broussais had now become an *à priori* medical philosopher: he had framed a creed of *rationalism*: he had established a new doctrine of his own: he was the acknowledged chief of a new party: a single dominant idea had taken possession of his mind. In this work, as in the other, the traces of his great genius are still evident. His greatness he could not put off, if he would: but the scientific rectitude of his mind is no longer present: the clearness of his vision has become obscured: the acute and circumspect observer of diseases, and their relationships, indifferent as to the result of his investigations, provided only, that this result was the expression of the actual truth, is now the interested seeker for certain particular phenomena, *which he wishes to find;* the upright and impartial judge has become the *ex parte* advocate and witness. And, as generally happens in similar circumstances, not only is his mind perverted by the influences of a false philosophy, but his passions are excited by the controversies which grow out of it. His arrogance and dogmatism are as offensive as his criticisms of those who refuse to follow him are injurious and unjust. The exigencies of his own creed led him into inconsistencies, and his contradictions of himself are as direct and flagrant, as they are humiliating. He has himself become an illustration of the reasonableness and propriety of one of his own sayings—'I hold it as a principle always to suspect the experience of a man whose mind is pre-occupied.' He remarks of Lord Bacon, that he often sacrificed at the altar of one of the idols which he had overthrown. And he too, it may be more truly said, redoubtable iconoclast as he is, has set up as false an idol as any which he has broken: and declared a vindictive and uncompromising warfare against all who refuse to fall down before it. And such are the natural, and almost inevitable results of a belief in any of those *à priori* systems." 210.

*Classification of Diseases.*—The author thus states his proposition upon this subject. "*Diseases, like all other objects of natural history, are susceptible of classification and arrangement, which will be natural and perfect, just in proportion to the number, the importance, and the degree of the similarities and the dissimilarities between the diseases themselves.*" The fundamental error which vitiates all existing classifications is, that they are founded upon the examination and comparison of only *certain particular and limited portions* of the phenomena and relationships. Generally certain striking symptoms of the disease are selected, omitting others often equally essential, and leaving unnoticed the morbid processes and alterations, which constitute the very diseases themselves. Additional impediments have arisen from the belief that the arrangement of diseases is capable of being as fixed and absolute as that of objects of physical science, thus producing a completely *artificial* system; and from the imperfect knowledge of the nature of many of them which prevailed at the period when these nosologies were constructed. The author proposes a more *natural* system, in which large classes of diseases may arrange themselves, according to their affinities, more or less nearly around certain centres, occupied by "type-species."

"One of the best and purest examples, one of the most perfect models, of such a family, is to be found in the exanthematous fevers. They constitute what may be called the *type family,* amongst these groups. Occupying the central region of this group, we find *small-pox, cow-pox, chicken-pox, measles,* and *scarlet-fever,* bound closely together, by numerous and very intimate affinities. They are all marked by the presence of that general morbid condition, designated by the term *fever;* they are characterized, each of them, by the presence of a peculiar cutaneous eruption; they are all self-limited, in duration; they pass through a regular series of processes, or changes, constituting so many distinct periods or stages; each of these diseases is capable of propagating itself, by means of a specific poison, or contagious principle; and, finally, they rarely affect the system more than once. At distances, farther and farther removed from this central position, we shall find the disease called *roseola, nettle-rash, erysipelas, plague, malignant pustule* and perhaps some others. These latter possess several of the characters, which belong to the former, but not all of them; and, as the affinities between them and the *type species* become fewer and feebler, they gradually recede from the central region which these occupy." 260.

Illustrations of similar arrangements into families are brought forward in fevers, inflammations, neuroses, &c.: the object of the author not being to frame a nosology, but to suggest the principles upon which it should be constructed. Of *Definitions* he says:—

"Although an adequate definition of a group, or family may be given in a few words, this is not often the case with a species, or a single member of one of these families. Such a definition must include *all the important and more constant phenomena and relationships* of the disease. It must be a clear and comprehensive enumeration of its elements. The omission of any one of these elements renders the definition, so far, inadequate, and defective."

After devoting a chapter to the consideration of the *relations of the chemical and vital forces*, in which he endeavours to place some curb upon the hastier generalizations of Liebig, the author terminates his work with a review of the *future prospects of medical science*. He considers that the direction the medical mind has taken during the last quarter of a century, holds out cheering prospects of future progress in the right direction. It is true that improvements in the *treatment* of disease have not, during the above-mentioned period, proceeded *pari passu* with our improved knowledge of the *nature* of disease; for the study of therapeutical relations is far more difficult and complex than that of the pathology and diagnosis of disease, and is perfected later than these, which are thus necessary preliminaries. Yet even the treatment of disease has made great advances, and will make yet greater, by reason of the great perfection of accurate *diagnosis* that has been arrived at. It is a matter of congratulation, too, that the employment of *heroic* remedies has become so moderated, and that a proper trust in the restorative powers of Nature is beginning to be felt. The axiom of Chomel that *to do good* is only the second law of therapeutics, the first being *not to do harm*, is fast making its way among us.

In estimating to whom will be due the future advancement of practical medicine, the author takes a brief review of the present position of medical science in France, Britain, and the United States. *France* he considers as justly entitled to the honor of having established the *modern school of medical observation*, with its minute analysis of morbid phenomena, and rejection of *à priori* reasonings. The works of Prost, Broussais, Corvisart, Laennec, Serres, Rostan, Lallemand, Rayer, Martinet, Chomel, Andral, Louis; and a host of other celebrated names, present a galaxy, of which that country may well be proud.

"Since the time of Hippocrates, there has not appeared in any age, or country, a series of cotemporaneous publications, upon similar subjects, at all equal to these in extent, variety, and positive value. There is hardly an important point in pathology upon which they have not shed new light—there is hardly a disease, the diagnosis of which they have not rendered easier and more certain, than it formerly was, while in many cases we are wholly indebted to them for our means of diagnosis: and they have added not a little to the exactitude of our knowledge in regard to some therapeutical processes."

The *British* school of observation commenced with Sydenham, and has undergone little change, having but slightly participated in the great continental movement of our times.

"This school has been marked by some of the strongest and best qualities of the British character—sagacity, shrewdness, and sound common sense. It has been regularly progressive since the time of Sydenham, and has accumulated a vast amount of most excellent practical knowledge. Its therapeutical resources have been more various and extensive, than those of its continental rival; and, if it has done less for the advancement of medicine *as a science*, it can hardly be doubted, I think, that it has accomplished more, as a useful and beneficent art. The principal defects of the British School are its want of comprehensiveness, of rigorous and positive conclusions, and the habit of mixing up, with its observations, reasonings and interpretations altogether hypothetical in their character; and then of regarding these reasonings as more important, more valuable, more essential, to the constitution of science, than the observations upon which they are founded."

Dr. Bartlett admits, however, that these blemishes are not universal and are in course of disappearing, and states that, although our modern medical observers have been too slow in adopting the rigorous numerical method of Louis (to whom he dedicates his work with a just eulogium), several of an older date pursued inquiry by means no less exact, as Percival, William Brown of Edinburgh, Woolcombe, John Cheyne, &c.

The medical mind in the *United States* seems to partake of the characters of that of both Britain and France. Prior to the publication of *Bichat's General Anatomy*, medical opinions were almost exclusively derived from Britain, and nearly every English work of importance has been republished and extensively read.

"The last 15 years, however, have witnessed a great change in this respect. While our medical relations with Britain are still, as they will always continue to be, numerous and intimate, they are altogether less exclusive than formerly; and they are now probably inferior, in interest, influence, and importance, to those which exist between us and France. Our young men have almost ceased to visit the British capitals, in order to complete their education; and the number of those who have repaired to Paris, for this purpose, for many years past, has been very much greater than has ever been the case with Edinburgh and London. The leading works of Louis were earlier and more widely circulated in this country than in Britain; and the principles of his school and method have taken deeper root here, than there." 306.

We believe the author has somewhat exaggerated both the amount of evil which the indulgence in hypothesis has exerted upon the progress of medical science, and the actual extent of its present influence in practice, especially the latter. So too, as before mentioned, he appears to us to misapprehend or confuse the ordinary acceptation of the process of reasoning by induction; and hence to seem in hostility with a mode of procedure he is really not only not adverse to, but an eloquent and able advocate in favour of. However this may be, we cordially recommend his work to the notice of our readers, as one of the highest utility in its general tendencies.

