# Errata

**Published:** 1845-01

**Authors:** 


					Xiffl"6.?-0, afle'' fhC W?rd " ?f'" insert " Practical medicine, grow out of
Pa J Jnn Vf-V obscu"ties. the uncertainties, the imperfections."

				

